# Relationship between polymorphism of receptor SCARB2 gene and clinical severity of enterovirus-71 associated hand-foot-mouth disease

**DOI:** 10.1186/s12985-021-01605-0

**Published:** 2021-06-30

**Authors:** Xia Wang, Hong Liu, Ying Li, Rui Su, Yamin Liu, Kunyan Qiao

**Affiliations:** Department of Childhood Infectious Diseases, Tianjin Second People’s Hospital, Tianjin, 300192 China

**Keywords:** Enterovirus 71, Human scavenger receptor B2, Single nucleotide polymorphism, Severe hand-foot-mouth disease

## Abstract

**Background:**

To investigate the relationship between polymorphism of scavenger receptor class B member 2 (SCARB2) gene and clinical severity of enterovirus (EV)-71 associated hand-foot-mouth disease (HFMD).

**Methods:**

Among the 100 recruited cases, 56 were in the severe HFMD group (case group) and 44 were in the general HFMD group (control group). By screening functional single nucleotide polymorphisms (SNPs) and hot SNPs, and performing SNP site optimization, some SNP sites of SCARB2 gene were selected for analysis. Genotyping was performed using a MassArray platform. PLINK software was used for statistical processing and analysis of the correlation differences between the mutant genotypes in the severe and general HFMD groups. The relationship between the SNPs and clinical severity of enterovirus (EV)-71 associated HFMD was assessed.

**Results:**

28 SNPs in SCARB2 were selected by site optimization. Then three loci were not in agreement with the minor allele frequency (MAF) in the 1000 Han Chinese in Beijing (CHB) dataset. Another three loci could not be detected. Nine loci were not suitable for further analysis (MAF < 0.01 and Hardy–Weinberg [HWE] *P* < 0.001). A total of 13 sites were subsequently analyzed. Through Fisher analysis, the frequency of the rs6812193 T allele was 0.134 and 0.034 in the severe and general HFMD groups, respectively (*P* 0.023 < 0.05, odds ratio [OR] 4.381 > 1). Logistic regression analysis of rs6812193 T alleles between the severe and general HFMD groups, respectively (*P* 0.023 < 0.05, OR 4.412 > 1, L95 1.210 > 1). Genotype logistic regression analysis of the rs6812193 alleles CT + TT versus CC gave an OR of 4.56 (95% confidence interval [95% CI] 1.22–17.04, *P* = 0.012).

**Conclusion:**

The rs6812193 T allele was a susceptibility SNP for SHFMD, and the rs6812193 polymorphism might be significantly associated with the susceptibility to EV-71 infection.

## Introduction

Hand, foot, and mouth disease (HFMD) is an infectious disease caused by a variety of enteroviruses which belongs to the small RNA virus family [1]. HFMD is common in children under 5 years of age, and it is mainly manifested as herpes and maculopapules on the hands, feet, mouth, and other areas. A few patients progress rapidly and develop neurogenic pulmonary edema, circulatory disturbance, and even death at 1–5 days after disease onset [2]. The following indicators should alert the clinician of possible deterioration and impending critical type of severe case: persistent high fever, nervous system involvement, abnormal respiratory rate and rhythm, circulatory dysfunction, elevated peripheral white blood cell count, elevated blood glucose, elevated blood lactic acid [3]. HFMD is a global disease with a variety of causes. The main causes of HFMD are enterovirus (EV)-71 and Coxsackievirus A16 infection; however, EV-71 is responsible for most of the severe cases and fatal cases [4].

EV-71 is another important neuroenterophilic virus after poliovirus elimination. An analysis shows that EV-71 was circulating in the Netherlands as early as 1963 [5], but it was first reported in 1969 [6]. It was global distribution and occasionally concentrated outbreak. Since 1997, outbreaks of HFMD caused by EV-71 have occurred in the Asia–Pacific region, such as Malaysia [7], Taiwan [8], Singapore [9], etc. In 1998, the epidemic outbreaks began in Chinese mainland, especially in 2007, Shandong province [10] and 2008 Anhui province [11], which resulted in a large number of severe and dead children. Therefore, in 2008, the Ministry of Health of China listed HFMD in the list of Class C infectious diseases. In recent years, the incidence of HFMD has been 37.01–205.06 per 100,000, with a fatality rate of 6.46–51.00 per 100,000 [3]. HFMD has the highest number of cases and deaths of all Class C infectious diseases in China and represents a serious threat to the health of children.

As a major cause of severe and fatal cases, the pathogenesis of EV-71 has attracted more and more researchers' attention. In clinical work, it is not difficult to find that the severity of clinical symptoms and prognosis of different children with the same infection with EV-71 are significantly different. As the first important portal for the virus to enter the human body, the virus receptor determines the host range of a virus and tissue specificity. The influence of individual differences on the severity of clinical symptoms is worth further studying. Yamayoushi et al. [12, 13] confirmed that SCARB2 is the receptor of all EV-71 strains in cell experiments. The guidelines for the diagnosis and treatment of HFMD issued by the Ministry of Health of China (2018) clearly indicate that SCARB2 is the main receptor of EV-71 virus. Studies have found that people with different genotypes and alleles have different probability of disease and severity [14]. Single nucleotide polymorphisms (SNPs) are the most common form of variation in human genomic DNA. The SNPs of SCARB2 gene have naturally attracted great attention. At present, most studies focus on the relationship between the severity of EV-71 infection and the polymorphism of cytokines such as TNF-ɑ, IL-6, IL-10 [15, 16], chemokine IP-10, MCP-1(CCl2) [17], immune-related factors OAS1, OAS2, OAS3 and MXA [18, 19]. To the best of our knowledge, few studies on the relationship between SCARB2 SNP and EV-71 HFMD have been performed. In this study, 28 SNP sites in SCARB2 were selected as study loci, expecting to further clarify the pathogenesis of EV71 infection, and to provide a strong research basis for the early warning of critical disease and the reduction of case fatality rate.

## Materials and methods

### Clinical data and sample collection

We recruited 100 children with HFMD admitted to our hospital from April 2018 to October 2020 who were positive after EV-71 nucleic acid test. Diagnosis was based on the “Guidelines for the Diagnosis and Treatment of Hand, Foot, and Mouth Disease” (2018) [3]. According to the occurrence and development process of the disease, HFMD is divided into general HFMD and severe HFMD. General HFMD is usually in the eruption stage, but severe HFMD includes nervous system involvement stage, early cardiopulmonary failure stage, and cardiopulmonary failure stage, based on the degree of danger and heavy. There were 56 cases with severe HFMD (as the case group) and 44 cases with general HFMD (as the control group). This study was approved by the Ethics Committee of Tianjin Second People's Hospital, and informed consent was obtained from the patients’ parents or family members.

HFMD samples were collected before treatment on the day of admission; 3 mL peripheral venous blood was collected and stored at − 80 °C for later use.

### SCARB2 SNP selection

#### Screening of SNPs

1. Screening of functional SNPs.

- In the National Center for Biotechnology Information (NCBI), SNP was searched using the name of the SCARB2 to identify functional SNPs including promoter proxy (upstream variant 2 KB), 5′-untranslated region (UTR), exons (missense, synonymous), 3′-UTR, etc. The relevant optimization parameter was a minor allele frequency (MAF) in Han Chinese in Beijing (CHB) > 0.05, according to the HapMap or 1000 Genomes databases.
- The screened SNPs were used for functional prediction.
- Linkage disequilibrium analysis was performed on the identified SNPs, and the linked sites with R^2^ = 1 were labeled.

2. Screening of hot SNPs.

- Through Google Scholar, the research literature of candidate gene-related polymorphisms was retrieved and susceptibility SNPs were screened out.
- The screened SNPs were verified using the CHB MAF values.

#### SNP site optimization method

According to the linkage disequilibrium analysis results, the completely linked loci with R^2^ = 1 were discarded, and the loci in the promoter region with R^2^ > 0.8 were retained (Haplotypes in this region are important for gene expression); however, the strong association sites with R^2^ > 0.8 identified in other regions and literature studies, are meaningless and omitted.

### DNA extraction

DNA was extracted from the blood samples using a Tiangen kit. DNA samples were analyzed on a NanoDrop2000, and 1.25% agarose gel electrophoresis was performed. DNA was quantified and transferred to a 96-well plate for storage at − 20 °C for later use.

### SNP typing

#### Primer design and synthesis

Assay Designer 3.1 software was used to design the primers, and the primers were synthesized by the company [BGI Tech Solutions (Beijing Liuhe) Co., Ltd]. The primer sequences are shown in Table 1.

**Table 1:** Primers used for SNP typing

SNP ID	2nd-PCRP	1st-PCRP	Extension of primers	Amplified fragment length (bp)
rs1051326	ACGTTGGATGAGTGAGTGACAGTGAGCTAC	ACGTTGGATGTATTTCTTCTGGGACAGCCG	GAGGAAGGAACTTGTAAAAA	127
rs11547135	ACGTTGGATGAGAGCTGCGCGCACGAACC	ACGTTGGATGATCCAACTGCAAGGAGGGAG	cccctCGCCGAAGGGTCCCG	125
rs121909118	ACGTTGGATGTGACCAGAGTCCACATTCAC	ACGTTGGATGACTTTATACCGAAAGGCAGG	TTTCAGTGACTATGAGAGTGTA	129
rs121909119	ACGTTGGATGGAGATATCGGGCCTGAAAAC	ACGTTGGATGCAGCAGAAGCTCTTTGTGAC	GATTTCATCTTTGTAGCCC	118
rs1465922	ACGTTGGATGAAGGAAACCGAAACCGAGTC	ACGTTGGATGTTCGTGCGCGCAGCTCTGG	taCCGGTGCACCCGGGG	126
rs1465923	ACGTTGGATGATCCCTAGGTTGCTGCAAAG	ACGTTGGATGTCCCAGAAGTCTGGCATCTC	atgcGCACAGCAGGGATACTAAGGC	131
rs1470194	ACGTTGGATGGGTTGGTGTAGGTGAATTAG	ACGTTGGATGACTGAAGCTTCTACCTCCTG	cccacGTTTACTGGGCCAGCCCAGG	136
rs200053119	ACGTTGGATGTCTGCTGTTAGCAACAAGGC	ACGTTGGATGGCCTACTTACCAATACAGGA	AACAAGGCCTATGTTTTTGAA	126
rs2119733	ACGTTGGATGCAAGTCTGAAACCCAACAGG	ACGTTGGATGACCAGTGTGCTCTGGATGTG	ACAGGGCAGTTATTAAATC	111
rs2869851	ACGTTGGATGATGCCAGTCACTGTCCTAAG	ACGTTGGATGAATACAAGCATGAGCCACCG	gttgACATATTTACATGTAGTTAATGC	139
rs3733255	ACGTTGGATGAAACTGTGTGAGCTGTCCTG	ACGTTGGATGGGCCAGAATGTTCCTATCAC	TGTTGAAAGAAGGAAAAAGACAC	145
rs3733256	ACGTTGGATGGGCCAGAATGTTCCTATCAC	ACGTTGGATGAAACTGTGTGAGCTGTCCTG	aTGCAAGGAGGTGGAG	145
rs57374265	ACGTTGGATGGTCAGGGTTCATCCATGTTG	ACGTTGGATGGTGGTATATCTACACAACGG	CATCCATGTTGTAGCATGTAT	105
rs6811781	ACGTTGGATGAGAGAGTCTCACTCTGTCGC	ACGTTGGATGTGGCTGAGGCAGGAGAATTG	gcttCAACCTCTGCCTCCC	120
rs6812193	ACGTTGGATGACTTGATCATGGACTCCACC	ACGTTGGATGTGCAGTGGTTAATAACATGG	GGGAAAGCTGGATTTGAA	112
rs6824953	ACGTTGGATGTCCCAATGTACTGGAAGCTC	ACGTTGGATGTAAACCACAGTTGAAGATG	gggttGGGTGCAGTGACCAAGTCCTTT	137
rs6841815	ACGTTGGATGTGGCTGAGGCAGGAGAATTG	ACGTTGGATGAGAGAGTCTCACTCTGTCGC	AGTGAGCCGAGATCA	120
rs727502772	ACGTTGGATGTCTGAGTCTGAAAACACCCG	ACGTTGGATGAGTGAAACGGGAGACATTAG	TCCGTCTCAGGACTTA	119
rs727502781	ACGTTGGATGGGACTACACAGAAATGGTGC	ACGTTGGATGGATTTGGAACCTCTTGGCTG	gccgTTTCACTTCTCTGATTTGC	149
rs72857048	ACGTTGGATGGGAGGAATCCTGTCTTTTAC	ACGTTGGATGTGATCATGCCACTGCATTCC	AAGTCTTGCTCTGTTGC	134
rs75285019	ACGTTGGATGTAGAGACTGCAGCTACTAAG	ACGTTGGATGGAGAAGACTCTATCCTAGGC	tgtTGGAGATCGAAGCTATAAT	106
rs755903502	ACGTTGGATGCTCTCTTCTGTGTTTCAGGG	ACGTTGGATGAGGACAGCTCACACAGTTTC	gggttTGTTTCAGGGAACAGCGGATG	118
rs7697073	ACGTTGGATGATCTGACTCCAAATCTCACG	ACGTTGGATGTAAGGTGTGATCTTTCTGGG	TCTAATAAAAATAAAGTTGCTATCAC	124
rs78737354	ACGTTGGATGATGCCTTGCAACTTCTGCTG	ACGTTGGATGATGTTCTCTGCAGCAGTCTC	acccGGAGCCTCAAGTCACC	118
rs886041074	ACGTTGGATGATGATCTCCCTGAGGAAGTG	ACGTTGGATGGGGATGCTGCTGTCTTAATA	actgtGACCACTCTATGACAGTC	100
rs886041076	ACGTTGGATGGACAGTTACCTTAACTTTAC	ACGTTGGATGCTGAAAAAATAATTCCACTG	tggGGAATGGAATGGGAAAAC	105
rs886041078	ACGTTGGATGTTTCTTGCCTCTCCAGAGTG	ACGTTGGATGGTAGGGTATGTTGGTGATG	AAAGAGACGGCGAGT	117
rs8475	ACGTTGGATGCATTTAACTAGATAATTGGGC	ACGTTGGATGCCTGATAATAGGACTAAACC	ggggAGATAATTGGGCATGTCTTA	125

#### Primer dilution and extension mix configuration

The single-tube PCR masters were diluted to 100 μM, and deionized water was added to achieve a final PCR master mix concentration of 0.5 μM. The single tube extension primers were diluted to a final concentration of 500 μM. Each primer was diluted to 8 μM, 10 μM, and 15 μM. According to the instructions of the DNA synthesis products, the molecular weight and number of moles, the amount of deionized water to be added were calculated according to the required concentration. According to the molecular weight of the mixed single-tube extension primers, 1 time (< 6300 Da), 1.2 times (6300–7200 Da), and 1.5 times (> 7200 Da) were taken for mixing.

#### MassArray reactions

PCR amplification was conducted in 5 µL reactions, containing 1.000 µL DNA (20 ng/μl), 1.000 µL PCR primers (500 nM each), 0.100 µL dNTP mix (25 mM each), 0.625 µL PCR buffer (15 mM MgCl_2_), 0.325 µL MgCl_2_ (25 mM), 1.850 µL water HPLC grade, and 0.100 µL Taq DNA polymerase (5 U/µL) (Agena Bioscience, San Diego, CA, USA). The PCR conditions were as follows: 94 °C for 5 min, 94 °C for 20 s, 45 cycles of 56 °C for 30 s, 72 °C for 1 min, and a final extension step at 72 °C for 3 min. Remaining unincorporated dNTPs were dephosphorylated and inactivated by treatment with 1 U shrimp alkaline phosphatase at 37 °C for 20 min and then 85 °C for 5 min. Finally, the single base extension reaction mix, including iPLEX Buffer Plus, iPLEX Termination mix, Extension Primers mix, and iPLEX enzyme (Agena Bioscience, San Diego, CA, USA), was added to the PCR amplification products. The single base extension reaction was carried out under the following conditions: 94 °C for 30 s, 40 cycles at 94 °C for 5 s (52 °C for 5 s and 80 °C for 5 s, repeated 5 times per cycle), and a final extension step at 72 °C for 3 min. The samples were spotted on a SpectroCHIP (Agena Bioscience, San Diego, CA, USA), and analyzed by mass spectrometry. The spectral profiles generated by matrix-assisted laser desorption/ionization-time of flight mass spectrometry were analyzed using Typer v.4.0 software (Agena Bioscience, San Diego, CA, USA).

### Statistical analysis

PLINK software was used for statistical processing and analysis of the correlation differences between the mutant genotypes in the case and control groups. Case: severe HFMD group; Control: general HFMD group. A1: mutant; A2: wild-type (the default is the variant with the lowest allele frequency). A1 frequency is the MAF value. According to the Hardy–Weinberg equilibrium, the selected samples were from a random population. Fisher test was used to compare the genotype frequency between the case group and the control group. P-value represents the statistical difference between both groups, *P* < 0.05 indicates that there is a significant difference in A1 between the case and control groups; OR < 1 indicates that A1 is protective; OR = 1 indicates that A1 has no relationship with disease; OR > 1 indicates that A1 has a pathogenic effect. The differences of alleles and genotypes were compared by Logistic regression analysis. 95% confidence interval = L95 – U95. L95 and U95 represent the lower and upper limits of the confidence interval, respectively. OR > 1 and L95 > 1 indicate that the allele has a pathogenic effect, while OR < 1 and U95 < 1 indicate that the allele has a protective effect.

## Results

### Characteristics

The characteristics of the subjects are shown in Table 2. There is no statistically significant difference between the case and control groups in terms of age and sex (*P* > 0.05).

**Table 2:** Characteristics of the subjects

	Characteristics	n (100)	Case	Control	χ^2^	*P* value
Age	> 3 years	36	17	19	1.769	0.185
	≤ 3 years	64	39	25		
Sex	Male	53	29	24	0.075	0.784
	Female	47	27	20		

### Optimized SNP sites

The selected 28 SNP sites in SCARB2 were: rs1051326, rs11547135, rs121909118, rs121909119, rs1465922, rs1465923, rs1470194, rs200053119, rs2119733, rs2869851, rs3733255, rs3733256, rs57374265, rs6811781, rs6812193, rs6824953, rs6841815, rs727502772, rs727502781, rs72857048, rs75285019, rs755903502, rs7697073, rs78737354, rs8475, rs886041074, rs886041076, and rs886041078.

Among the 28 optimized sites, the MAFs for rs6811781, rs6841815, and rs72857048, were not in agreement with those in the 1000 CHB dataset, so it was discarded. rs11547135, rs1465922, and rs2869851 could not be detected so that they were excluded from further analysis. Of the 28 SNPs examined, consideration was given to the accuracy of the reaction system and primers. Therefore, SNPs with low detection rates were not used in further analysis. Therefore, a total of 22 SNPs were analyzed further.

The MAFs of the selected SNPs were greater than 0.01, and the P-values of the Hardy–Weinberg equilibrium test were greater than 0.001. Nine SNPs (rs121909118, rs121909119, rs200053119, rs727502772, rs727502781, rs755903502, rs886041074, rs886041076, and rs886041078) did not fulfill these criteria and were excluded from further analysis. At last, a total of 13 sites were subsequently analysed (Table 3).

**Table 3:** Genotyping results

		Allele N		Genotype N (%)				MAF (A1)	H–W *P* value
		A1	A2				Undetected		
rs1051326		C	G	CC 21	GG 39	CG 35	5		
	Case	49	59	14	19	21	2	0.405	0.021
	Control	28	54	7	20	14	3		
rs1465923		C	T	CC 0	TT 92	TC 8			
	Case	5	107	0	51	5		0.040	1.000
	Control	3	85	0	41	3			
rs1470194		C	A	CC 8	AA 51	CA 41			
	Case	31	81	2	27	27		0.285	1.000
	Control	26	62	6	24	14			
rs2119733		A	T	AA 0	TT 97	TA 3			
	Case	2	110	0	54	2		0.015	1.000
	Control	1	87	0	43	1			
rs3733255		T	C	TT 0	CC 92	TC 8			
	Case	6	106	0	50	6		0.040	1.000
	Control	2	86	0	42	2			
rs3733256		C	G	CC 0	GG 92	CG 8			
	Case	6	106	0	50	6		0.040	1.000
	Control	2	86	0	42	2			
rs57374265		A	G	AA 13	GG 36	GA 51			
	Case	47	65	8	17	31		0.385	0.529
	Control	30	58	5	19	20			
rs6824953		C	G	CC 6	GG 43	GC 50	1		
	Case	31	79	3	27	25	1	0.313	0.106
	Control	31	57	3	16	25			
rs75285019		A	G	AA 0	GG 92	AG 8			
	Case	6	106	0	50	6		0.040	1.000
	Control	2	86	0	42	2			
rs7697073		C	T	CC 16	TT 36	CT 48			
	Case	47	65	11	20	25		0.400	1.000
	Control	33	55	5	16	23			
rs78737354		T	C	TT 13	CC 46	CT 41			
	Case	39	73	6	23	27		0.335	0.500
	Control	28	60	7	23	14			
rs8475		A	T	AA 13	TT 36	TA 51			
	Case	47	65	8	17	31		0.385	0.529
	Control	30	58	5	19	20			
rs6812193		T	C	TT 1	CC 83	CT 16			
	Case	15	97	1	42	13		0.090	0.568
	Control	3	85	0	41	3			

Undetected: locus detection rate > 95%, which meets the requirements for locus detection

### Fisher analysis

As shown in Table 4, the frequencies of the rs6812193 T allele was 0.134 and 0.034 in the case and control group, respectively. *P *value 0.023 < 0.05, indicating a significant difference of A1 between the case and control groups; the OR of 4.381 > 1 indicates that A1 has a pathogenic effect. The remaining 12 SNPs may not be related to the pathogenicity of EV-71. Therefore, the rs6812193 T genotype is a susceptibility SNP.

**Table 4:** Fisher analysis

SNP	A1	FA	FU	*P* value	OR
rs1051326	C	0.454	0.342	0.137	1.602
rs1465923	C	0.045	0.034	1.000	1.324
rs1470194	C	0.277	0.296	0.875	0.913
rs2119733	A	0.018	0.011	1.000	1.582
rs3733255	T	0.054	0.023	0.470	2.434
rs3733256	C	0.054	0.023	0.470	2.434
rs57374265	A	0.420	0.341	0.306	1.398
rs6824953	C	0.282	0.352	0.355	0.722
rs75285019	A	0.054	0.023	0.470	2.434
rs7697073	C	0.393	0.409	0.885	0.935
rs78737354	T	0.348	0.318	0.763	1.145
rs8475	A	0.420	0.341	0.306	1.398
rs6812193	T	0.134	0.034	0.023	4.381

*FA* case group A1 allele frequency, *FU* control group A1 allele frequency

### Allele logistic regression analysis

As shown in Table 5, the *P* value of the rs6812193 T allele was 0.0245 < 0.05, indicating a significant difference between the case and control groups; the OR of 4.412 > 1 and L95 value 1.210 > 1 indicate that the allele had a pathogenic effect. The remaining 12 SNPs may not be related to the pathogenicity of EV-71. Therefore, the rs6812193 T genotype is a susceptibility SNP.

**Table 5:** Allele logistic regression analysis

SNP	A1	L95	U95	STAT	*P* value	OR
rs1051326	C	0.856	2.520	1.395	0.163	1.469
rs1465923	C	0.302	5.941	0.385	0.700	1.340
rs1470194	C	0.491	1.695	– 0.291	0.771	0.912
rs2119733	A	0.140	18.160	0.375	0.708	1.593
rs3733255	T	0.483	13.150	1.097	0.273	2.520
rs3733256	C	0.483	13.150	1.097	0.273	2.520
rs57374265	A	0.784	2.651	1.176	0.240	1.441
rs6824953	C	0.342	1.315	– 1.162	0.245	0.671
rs75285019	A	0.483	13.150	1.097	0.273	2.520
rs7697073	C	0.529	1.652	– 0.233	0.816	0.935
rs78737354	T	0.640	2.009	0.430	0.668	1.134
rs8475	A	0.784	2.651	1.176	0.240	1.441
rs6812193	T	1.210	16.080	2.249	0.0245	4.412

### rs6812193 genotype logistic regression analysis

As shown in Table 6, in the dominant model, the rs6812193 T allele was associated with a risk of severe disease. CT + TT genotype carriers had an increased risk of severe disease compared with CC genotype carriers (OR = 4.56, 95% confidence interval = 1.22–17.04, *P* = 0.012).

**Table 6:** rs6812193 genotype logistic regression association with response status (n = 100)

Model	Genotype	Status = 1	Status = 2	OR (95% confidence interval = L95–U95)	*P* value
Codominant	C/C	41 (93.2%)	42 (75%)	1	0.035
	C/T	3 (6.8%)	13 (23.2%)	4.23 (1.12–15.95)	
	T/T	0 (0%)	1 (1.8%)	NA (0.00-NA)	
Dominant	C/C	41 (93.2%)	42 (75%)	1	0.012
	C/T-T/T	3 (6.8%)	14 (25%)	4.56 (1.22–17.04)	
Recessive	C/C–C/T	44 (100%)	55 (98.2%)	1	0.28
	T/T	0 (0%)	1 (1.8%)	NA (0.00-NA)	
Overdominant	C/C-T/T	41 (93.2%)	43 (76.8%)	1	0.021
	C/T	3 (6.8%)	13 (23.2%)	4.13 (1.10–15.56)	

*Codominant* TT versus CT versus CC, *Dominant* (CT + TT) versus. CC, *Recessive* TT versus (CT + CC), *Overdominant* (CC + TT) versus CT, *NA* not applicable

## Discussion

The human SCARB2 gene is located on chromosome 4 and encodes a peptide chain containing 478 amino acids. SCARB2 is a transmembrane sialic acid glycoprotein with a relative molecular mass of 85 kDa, and belongs to the family of CD36 molecules [20]. SCARB2 is mainly located in lysosomes and endosome, and widely present on the membrane of most human cells including nerve cells [21]. This protein is also called lysosomal integral membrane protein 2. It is a type of specific glucose cerebral fat enzyme combined with ligands, involved in the lysosomal pathway. The related research fields are mostly Parkinson's disease with abnormal lysosomal metabolism [22, 23], Gaucher’s disease and myoclonic epilepsy [24, 25]. Yamayoshi et al. [12, 13] found that the tissue distribution of EV-71 virus antigen was well correlated with SCARB2, and further found that this receptor was involved in the endocytosis and membrane transport of pathogenic bacteria. This study speculated that the expression level of SCARB2 might be related to virus sensitivity and infection rate. Therefore, the 28 selected sites were all functional sites related to the expression level, including exons, promoters and introns.

Choi M et al. [26] found that exons contain the vast majority of protein coding synthesis, and about 85% of pathogenic mutations are located in the exon region. In 2009, Ng SB et al. [27] used exome sequencing for the first time to find point mutations located in MYH3 in 4 patients with Freeman Sheldon syndrome (autosomal dominant genetic disease), showing the powerful effect of exome sequencing in identifying pathogenic genes of Mendelian genetic disease. Many complex diseases have been identified by exome sequencing, such as genetic disease OHDO syndrome (KAT6B) [28], CTNNB1 mutation in craniopharyngioma patients [29], point mutation of dilated cardiomyopathy GATAD1 [30], etc. Jenny Do et al. [25] found that 3'-UTR mutations in SCARB2 may be associated with Gaucher disease and myoclonic epilepsy. Yock-Ping Chow et al. [31] found that SCARB2 exon mutation was associated with Pendred syndrome. Yamayoshi et al. [13] found that amino acids at position 142-204 of SCARB2 played an important role in promoting the binding of virus particles to cells and susceptibility to EV-71. However, the study of Ting-Yu Yen et al. did not find the correlation between amino acids at position 142-204 and clinical severity [32]. There are 12 exon sites selected in this study: rs1051326, rs3733255, rs3733256, rs6811781, rs6841815, and rs8475 belong to 3' UTR region, and its function was predicted as miRNA binding site. rs11547135 and rs1465922 belong to 5' UTR region, and its function was predicted to be a TFBS transcription factor binding region. rs121909118 rs200053119 rs755903502 and rs886041078 are NCBI pathogenic clinical significance sites. Among these exons, rs6811781 and rs6841815 (not in CHB); rs11547135 and rs1465922 (not detected); rs121909118, rs200053119, rs755903502, rs886041078 (not in line with MAF value). Finally, rs8475, rs1051326, rs3733255 and rs3733256 were included in the study, but no correlation was found between these four exon loci and the severity of clinical infection.

Promoter is an important *cis*-element in gene expression regulation and the core region of gene transcriptional regulation. In this study, two promoter loci were selected: rs1465923 and rs78737354. Finally, no correlation between these two promoters and clinical infection was found.

Previously, it was often believed that introns do not encode proteins and do not have biological functions in organisms. However, studies have found that the expression profiles of the same gene with and without introns are significantly different [33]. In many cases of transgenic expression, the addition of a universal intron to cDNA results in a significant increase in gene expression [34, 35]. The optimal expression of many endogenous genes has been demonstrated in mammalian tissue culture cells, transgenic mice, insects, and plant systems requiring the presence of one or more introns. Therefore, a variety of introns in organisms are an important part of eukaryotic genome and are closely related to the construction and dynamic changes of cytoskeleton of gene expression [36]. Ting-Yu Yen studied the relationship between SCARB2, PSGL-1, ANXA2 polymorphisms and clinical severity, and found that rs11097262 was associated with rs6824953 located in the intron region of SCARB2 gene, considering that it may regulate the function or expression of SCARB2 and thus affect the susceptibility to EV-71 [32]. In this study, 14 introns were selected: rs121909119, rs727502772, rs727502781, rs886041074, and rs886041076 were considered to be the sites with pathological clinical significance on NCBI website; rs6812193 is a hot spot site that can be found in the literatures [23, 37–39]; rs1470194, rs2119733, rs2869851, rs57374265, rs6824953, rs72857048, rs75285019, and rs7697073 are the TAGSNP sites. Among these introns of this study, rs72857048 is not in CHB; rs2869851 is not detected; rs121909119, rs727502772, rs727502781, rs886041074, and rs886041076 are not in line with MAF value. Finally, rs1470194, rs2119733, rs57374265, rs6812193, rs6824953, rs75285019 and rs7697073 were included in the final study, but only rs6812193 was correlated with the severity of clinical infection, and no correlation was found for the other 6 introns. By using Fisher analysis and allele logistic regression analysis, the rs6812193 T allele was shown to have a pathogenic effect. rs6812193 genotype logistic regression analysis in a dominant model showed that CT + TT genotype carriers had an increased risk of severe HFMD compared with CC genotype carriers. Therefore, the rs6812193 T genotype is considered to be a susceptibility SNP, and the rs6812193 polymorphism may be related to susceptibility to EV-71.

rs6812193 is actually a hot SNP close to SCARB2. A 2011 Web-based Genome-wide Association (GWA) study found that a nucleotide polymorphism rs6812193 close to SCARB2 was significantly associated with Parkinson’s disease (PD) in people of European ancestry [37]. In 2012, Shuai Chen et al. conducted a genotyping study on rs6812193 in 449 PD patients and 452 control patients in mainland China, and found that there is no statistically significant differences in allele and genotype distribution between the patients and the control group [38]. In 2013, Kallirhoe Kalinderi et al. studied 210 Greek patients with sporadic PD and 133 control subjects in Greece. It was found that there was no difference in genotype or allele frequency between PD patients and controls [39]. In 2021, T.S. Usenko et al. rs6812193 of the SCARB2 gene does not confer a significant risk for PD in Russian population [23]. At present, to the best of our knowledge, there is no research related to the role of rs6812193 in HFMD. How does rs6812193 affect the expression and function of SCARB2? This will be examined in the future by increasing the sample size to verify the association of rs6812193 with susceptibility to EV-71 HFMD, and to study the function of rs6812193.

The present study also has some limitations, such as the small number of cases, which may make it difficult to find significant differences between low-frequency SNPs. In addition, with sufficient funds and time, genome-wide tests can be performed to avoid screening for missing sites of interest. Clarifying the relationship between gene polymorphisms and disease will enable us to analyze disease pathogenesis further, to explore the nature of the diversity of disease phenotypes, and to develop more individualized treatment measures.

## Conclusion

The rs6812193 T genotype was identified as a susceptibility SNP. CT + TT genotype carriers have an increased risk of severe HFMD compared with CC genotype carriers. Therefore, the rs6812193 polymorphism might be considerably related to clinical severity of enterovirus (EV)-71 associated HFMD, which can support doctors to make evidence-based health recommendations to patients.

## Data Availability

The data used and/or analyzed during this study are available from the corresponding author on request.

## Abbreviations

SCARB2: scavenger receptor class B member 2
EV: enterovirus
HFMD: hand-foot-mouth disease
SHFMD: severe hand-foot-mouth disease
SNP: single nucleotide polymorphism
MAF: minor allele frequency
CHB: Han Chinese in Beijing
RNA: ribonucleic acid
DNA: deoxyribonucleic acid
HWE: Hardy–Weinberg
NCBI: National Center for Biotechnology Information
UTR: untranslated region
PCR: polymerase chain reaction
PCRP: polymerase chain reaction primers
HPLC: high performance liquid chromatography
dNTP: deoxy-ribonucleoside triphosphate
A1: mutant
A2: wild-type
FA: case group A1 allele frequency
FU: control group A1 allele frequency
OR: odds ratio
L95: lower limits of the confidence interval
U95: upper limits of the confidence interval
STAT: coefficient t-statistic
MYH3: myosin heavy chain 3
KAT6B: lysine acetyltransferase 6B
GATAD1: GATA zinc finger domain containing 1
TFBS: transcription factor binding site
PSGL-1: P-selectin glycoprotein ligand 1
ANXA2: annexin II
GWA: Genome-wide Association
PD: Parkinson’s disease
